# Supplementary material for: The Construction and Exploration of a Comprehensive MicroRNA Centered Regulatory Network in Foxtail Millet (Setaria italica L.)
Source: Front Plant Sci. 2022 May 6;13:848474. doi: 10.3389/fpls.2022.848474 (PMC9121102; doi:10.3389/fpls.2022.848474)
Supplement: Supplementary file 1 [file Data_Sheet_1.zip › Sit-miR2118a_Seita.9G102600.1_827_TPlot.pdf]

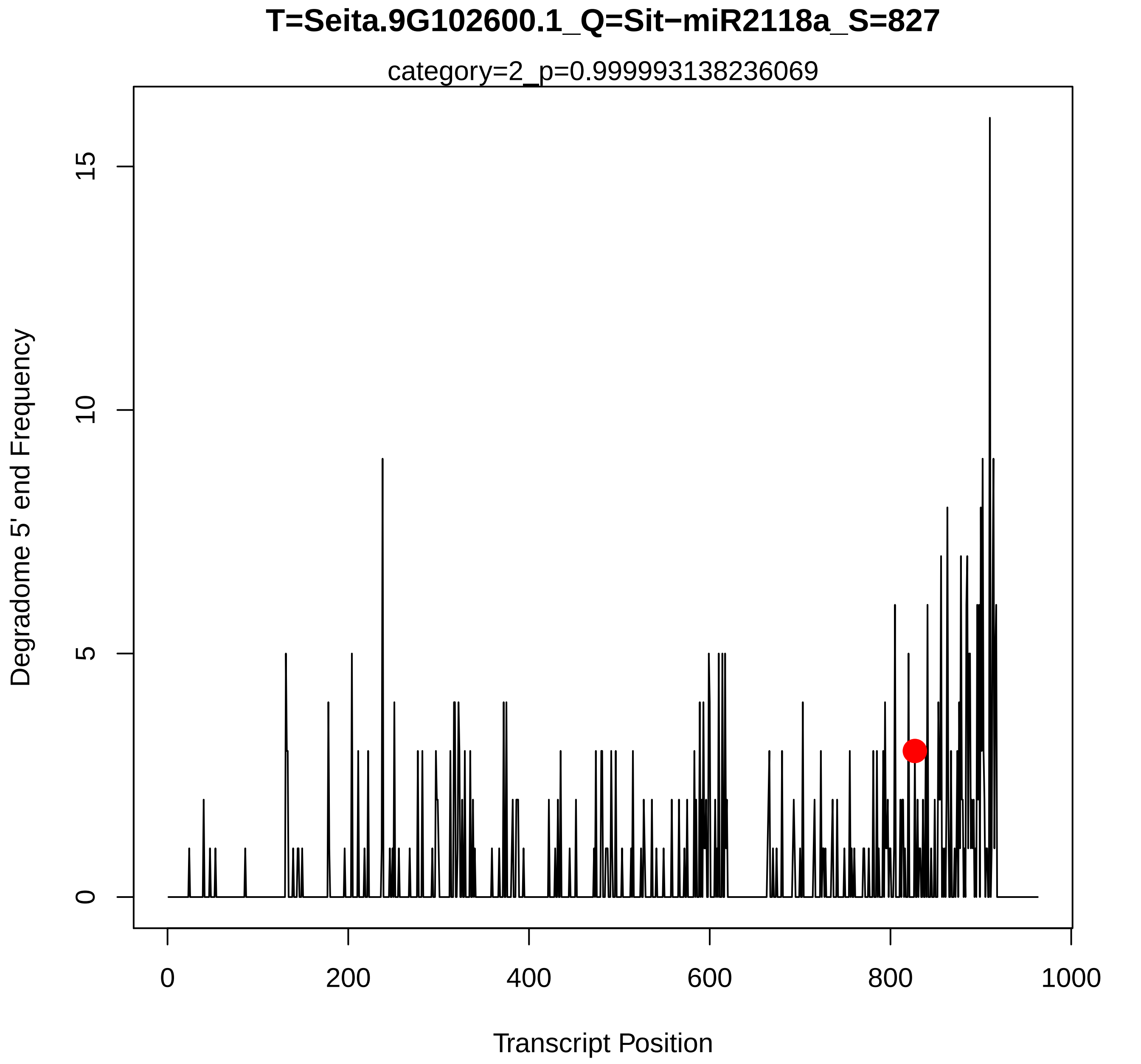
T=Seita.9G102600.1_Q=Sit−miR2118a_S=827
category=2_p=0.999993138236069
Degradome 5' end Frequency
Transcript Position
0
5
10
15
0
200
400
600
800
1000
